# Effects of thromboprophylaxis on mesenchymal stromal cells during osteogenic differentiation: an in-vitro study comparing enoxaparin with rivaroxaban

**DOI:** 10.1186/s12891-016-0966-2

**Published:** 2016-03-01

**Authors:** Hakan Pilge, Julia Fröbel, Silvia J. Mrotzek, Johannes C. Fischer, Peter M. Prodinger, Christoph Zilkens, Bernd Bittersohl, Rüdiger Krauspe

**Affiliations:** Department of Orthopaedic Surgery, Heinrich-Heine-University, Moorenstr. 5, 40225 Düsseldorf, Germany; Institute for Transplantation Diagnostics and Cell Therapeutics, Heinrich-Heine-University, Moorenstr. 5, 40225 Düsseldorf, Germany; Department of Orthopaedic Surgery, Klinikum rechts der Isar, Technical University Munich, Ismaninger Str. 22, 81675 Munich, Germany

**Keywords:** Rivaroxaban, Enoxaparin, MSC, Osteogenic differentiation, Bone healing

## Abstract

**Background:**

Low-molecular-weight heparins (e.g. Enoxaparin) are widely used to prevent venous thromboembolism after orthopaedic surgery, but there are reports about serious side effects including reduction in bone density and strength. In recent years new oral antithrombotic drugs (e.g. direct Factor Xa-inhibitor, Rivaroxaban) have been used to prevent venous thromboembolism. However, there is lack of information on the effects of these new drugs on human mesenchymal stromal cells during osteogenic differentiation and, therefore, effects during postoperative bone healing.

**Methods:**

We evaluated the effects of Rivaroxaban and Enoxaparin on the proliferation, mRNA and surface receptor expression as well as differentiation capacity of primary human mesenchymal stromal cells during their osteogenic differentiation.

**Results:**

Enoxaparin, but not Rivaroxaban treatment significantly increased human mesenchymal stromal cell (hMSC) proliferation during the first week of osteogenic differentiation while suppressing osteogenic marker genes, surface receptor expression and calcification.

**Conclusions:**

This is the first paper to demonstrate that Rivaroxaban had no significant influence on hMSC differentiation towards the osteogenic lineage, indicating a less affected bone healing process compared with Enoxaparin in vitro. Based on these findings Rivaroxaban seems to be superior to Enoxaparin in early stages of bone healing in vitro.

**Electronic supplementary material:**

The online version of this article (doi:10.1186/s12891-016-0966-2) contains supplementary material, which is available to authorized users.

## Background

Following general and orthopaedic surgery, thromboprophylaxis is essential to avoid venous thromboembolism (VTE). Current treatment options for prevention include heparin and low-molecular-weight heparins (LMWHs). Without any prophylaxis, the risk of developing VTE after major lower extremity orthopaedic surgery is between 40 and 60 % [1, 2]. With the use of Enoxaparin (a commonly used LMWH), the risk can be reduced to 2 % [3].

After binding to antithrombin III (AT3), Enoxaparin accelerates the activity of AT3 and potentiates the inhibition of factors Xa and IIa. As factor Xa catalyzes the conversion of prothrombin to thrombin, enoxaparin decreases thrombin and therefore ultimately prevents fibrin clot formation.

Until now, heparin and LMWHs have been the “gold standard”, although there are reports about adverse effects. Studies report that long-term administration negatively affect postoperative bone healing, increase the risk of fracture and of developing osteoporosis [4–7].

In recent years oral “factor Xa inhibitor” drugs, e.g. Rivaroxaban, have been used to prevent postoperative VTE. These drugs act directly upon factor Xa in the coagulation cascade and inhibit both thrombin formation and the development of thrombi and have no effect on platelets [8]. With Rivaroxaban, the occurrence of major VTE after hip arthroplasty was reduced to 0.2 % [3]. Furthermore, the incidence of major bleeding did not differ significantly between patients treated with Rivaroxaban (0.3 %) or Enoxaparin (0.1 %).

Enoxaparin was shown to inhibit cell proliferation at a prophylactic dose [9] and it is assumed that LMWHs alter the expression of cytokines (IL1, IL6, IL11 and tumor necrosis factor-α (TNF-α)) that are necessary for differentiation of MSCs into osteoblasts [10].

Interestingly, to date little is known about the cellular effects of oral factor Xa inhibitors on the migration, proliferation, differentiation and calcification of human mesenchymal stromal cells (hMSC) and osteoblasts. In a rat femur fracture model Rivaroxaban had no influence on fracture healing as revealed by micro CT-scans and biomechanical testing [11].

However, it is not known whether treatment with Rivaroxaban modulates postoperative bone healing in human patients. The aim of this study was to evaluate the effects of both Rivaroxaban and Enoxaparin on hMSCs during their osteogenic differentiation in vitro.

## Methods

The study protocol was approved and authorized by the Institutional Review Board according to the Helsinki Declaration. Informed consent was obtained from all patients before surgery. During elective surgery, bone marrow was harvested from the iliac crest (to fill osseous defects) or the femoral head (during elective hip-arthroplasty) of nine (five male, four female) patients aged 32 (±7) years. None of the patients had a history of bone marrow pathologies.

### MSC isolation and expansion

Bone marrow was filtered through 100 μm cell strainers (BD Biosciences) to separate cells from bone particles. Mononuclear cells were separated by density gradient centrifugation (Biocoll 1.077 g/ml, Biochrom) and washed with PBS. Erythrocytes were lysed using cold ammonium chloride solution. After counting the cells were seeded in growth medium (DMEM low glucose [Sigma-Aldrich], 20 % FBS Superior [Biochrom], 1 % penicillin/streptomycin/L-glutamine [Sigma-Aldrich]) in a humidified atmosphere at 37 °C and 5 % CO_2_. After 1 week, non-adherent cells were removed and growth medium changed every 3–4 days. Plastic-adherent mesenchymal stromal cells (MSC) were passaged weekly and seeded at 5000 cells/cm^2^. To fulfil the criteria of the International Society for Cellular Therapy and to exclude contamination of MSC cultures by hematopoietic cells MSC were analysed for a ≥95 % expression of CD73, CD90, CD105 while lacking CD34 and CD45, (method description below) and all experiments were carried out using MSCs derived from passage 3.

### Osteogenic differentiation

For osteogenic differentiation the hMSCs were seeded in 12-well plates (3.9 cm^2^ per well) or tissue culture flasks (75 cm^2^ per flask, both Greiner Bio-One) at 4000 cells/cm^2^ and cultured for 7, 14 and 21 days in differentiation medium (growth medium with 100 nM dexamethasone, 50 μM ascorbic acid and 20 mM β-glycerol phosphate; all Sigma-Aldrich) with medium changed twice weekly. For each time point there were flasks and wells with three different concentrations of Rivaroxaban (20, 100, 500 ng/ml) and Enoxaparin (2, 10, 50 μg/ml) spanning the median serum concentrations measured in former studies [12–14] as well as negative controls containing 0.1 % DMSO in which Rivaroxaban had to be solved or PBS, respectively.

### Cell count

After 7, 14 and 21 days in differentiation medium cells were trypsinized (Trypsin-EDTA, Sigma-Aldrich) and treated with a cell scraper (Greiner Bio-One). Cell count and viability were determined by haemocytometer using trypan blue exclusion and proliferation rate was calculated for each drug concentration and control flask, respectively.

### Quantitative real-time PCR

RNA was purified using RNeasy Mini Kit in combination with RNAse-free DNase kit, cDNA was synthesized by QuantiTect Reverse Transcription Kit according to the manufacturer’s instructions (all Qiagen). C_t_ values were measured in duplicate on a StepOne Real-Time PCR System using the SYBR Green PCR Master Mix (both Applied Biosystems). Glyceraldehyde 3-phosphate dehydrogenase (GAPDH) served as the reference control and its Ct values during drug treatment are provided in Additional file 1: Figure S1. Fold changes of mRNA expression levels were calculated using the 2^-ΔΔCt^ method [15]. Each drug-treated group was compared to its carrier control (DMSO for Rivaroxaban, PBS for Enoxaparin). Primers for ALPL, BGLAP, BMP2, CDH11, DKK1, RUNX2, OSX/SP7 and GAPDH were self-designed and purchased from Biolegio (Nijmegen, Netherlands). All other primers were QuantiTect Primer Assays purchased from Qiagen. Detailed sequence and purchase information is provided in Additional file 2: Table S1.

### Flow cytometric analysis of surface receptors

Fluorescence-conjugated antibodies (unless otherwise noted all BD Biosciences) against human CD10 (clone HI10a), CD34 (8G12), CD45 (2D1), CD49e (IIA1), CD73 (AD2), CD90 (5E10), CD92 (VIM-15b, Acris) and CD105 (266) as well as isotype controls (MOPC-21, 27–35) were used for flow cytometric staining. Cells were resuspended in Isoton® II Diluent (Beckman Coulter) and incubated with the antibodies for 15 min at room temperature in the dark. After 5 min fixing and washing in 0.1 % formaldehyde (Polysciences Europe) the flow cytometric analyses were performed using a FACSCalibur (BD Biosciences) and data were analysed with FCS Express V3 software (De Novo Software).

### Osteogenic staining

After 7, 14 and 21 days of osteogenic differentiation, respectively, medium was removed from the wells and cells were washed twice using PBS before a 5 min fixation in 4 % phosphate-buffered formaldehyde (Carl Roth). Staining of alkaline phosphatase (ALP) activity in the fixed cells was performed using the Vector Blue Alkaline Phosphatase Substrate Kit according to the manufacturer’s instructions (Vector Laboratories). Calcifications were stained using Alizarin red S (40 mM, pH 4.1, Carl Roth) for 20 min at room temperature with constant agitation. Microscopic images were taken using a stereomicroscope SteREO Discovery.V8 with an Achromat S 0.3x FWD 236 mm objective and processed using AxioVision software version 4.8.1 (all Carl Zeiss). Differentiation was quantified by measuring the intensity of alkaline phosphatase-stained cells in blue and Alizarin-stained calcified areas in red using ImageJ software (rsbweb.nih.gov).

### Statistical analyses

Statistical analyses were performed using GraphPad Prism (version 5.01, GraphPad Software Inc., CA, USA). Data are given as mean ± standard error of the mean (SEM). Kolmogorov-Smirnov tests for sample distribution and Friedman one-way ANOVA for paired samples with post-hoc Dunn’s multiple comparison were used for statistical examination. Adjusted p-values are provided in the text, and asterisks are used throughout the figures to indicate the levels of significance (*p < 0.05, **p < 0.01, ***p < 0.001).

## Results

### Effects on osteoprogenitor cell number

**Fig. 1:**
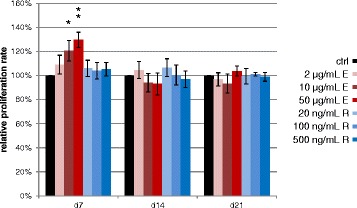
Effects on osteoprogenitor proliferation. Bar chart of cell count analysis showing the Enoxaparin dose-dependent increase in proliferation during the first week of osteogenic differentiation. Asterisks show significance levels of Dunn’s multiple comparison post-hoc tests to the control group (*n* = 9)

During osteogenic differentiation of the MSCs of each of the 9 donors, Enoxaparin treatment increased number of viable hMSCs during the first week in a dose-dependent manner (2 μg/ml: 109 %, 10 μg/ml: 121 %, 50 μg/ml: 130 %; *p* 0.145, 0.025, 0.002; respectively, Fig. 1). During the second and third week of differentiation, however, there were no measurable changes in the cell count of Enoxaparin-treated cells. Rivaroxaban treatment had no significant influence on the proliferation of the differentiating cells at any time (Fig. 1).

### Effects on osteoprogenitor mRNA expression

**Fig. 2:**
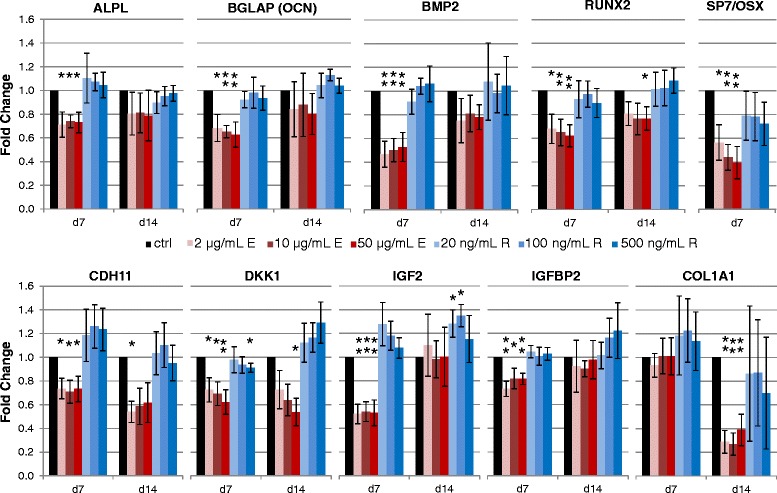
Effects on osteoprogenitor mRNA expression. Bar charts of relative mRNA expression of several osteogenic marker genes during differentiation showing the dose- and time-dependent influence of Enoxaparin. Asterisks show significance levels of Dunn’s multiple comparison post-hoc tests to the control group (*n* = 9)

RT-PCR analyses of the differentiating MSCs of each of the 9 donors showed that the expression levels of marker genes involved in osteogenic differentiation like alkaline phosphatase, liver/bone/kidney (ALPL), osteocalcin (BGLAP/OCN), bone-morphogenetic protein 2 (BMP2), Runt-related transcription factor 2 (RUNX2) and bone-specific transcription factor Sp7, also known as osterix (SP7/OSX), were significantly down-regulated after 7 days of Enoxaparin treatment while Rivaroxaban-treated cells showed no significant changes (Fig. 2). Furthermore, in the Enoxaparin-treated group we measured significantly lower expression levels of the osteoblast-specific cadherin (CDH11) and the Wnt signaling inhibitor Dickkopf-1 (DKK1) after 7 and 14 days of differentiation. IGF2 and its binding protein 2 (IGFBP2) were also down-regulated by Enoxaparin treatment after 7 days and collagen type I (COL1A1) after 14 days of differentiation. The only significant effects Rivaroxaban treatment had on the gene expression of the tested markers was a reduction of DDK1 on day 7 in cells treated with the highest concentration of the drug and an up-regulation of IGF2 after 14 days of differentiation (Fig. 2).

### Effects on osteoprogenitor phenotype

**Fig. 3:**
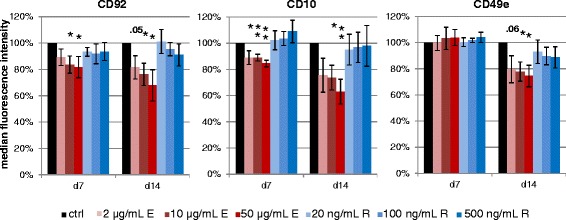
Effects on osteoprogenitor phenotype. Bar charts of surface receptor expression during osteogenic differentiation showing the dose- and time-dependent influence of Enoxaparin. Asterisks show significance levels of Dunn’s multiple comparison post-hoc tests to the control group (*n* = 9)

We further analysed the expression of several surface receptors of the MSCs of each of the 9 donors during osteogenic differentiation by flow cytometry. Whereas mesenchymal stromal markers CD73, CD90, CD105 and haematopoietic markers CD34, CD45 were not altered by treatment with either drug (data not shown), there were clear effects on three surface markers recently shown to be highly regulated during osteogenic differentiation [16]. As shown in Fig. 3 Enoxaparin treatment significantly down-regulated the surface expression of CD92 in a dose- and time-dependent manner (day 7: 2 μg/ml: 89 %, 10 μg/ml: 84 %, 50 μg/ml: 82 %, *p* 0.074, 0.027, 0.038; day 14: 82, 76, 68 %, *p* 0.053, 0.034, 0.037, respectively). The same effect was seen on CD10 expression (day 7: 89, 89, 85 %, *p* 0.041, 0.003, 0.001; day 14: 75, 74, 63 %, *p* 0.066, 0.025, 0.009, respectively). CD49e expression was not altered during the first week, but significantly decreased after 2 weeks of Enoxaparin treatment (80, 78, 74 %, *p* 0.059, 0.028, 0.018, respectively). Rivaroxaban had no significant effect on the expression level of these receptors during osteogenic differentiation (Fig. 3).

### Effects on osteogenic differentiation capacity

**Fig. 4:**
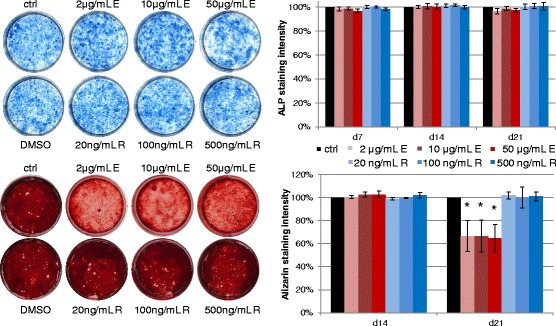
Effects on osteogenic differentiation capacity. Representative microscopic images out of 5 hMSC cultures with alkaline phosphatase staining after 14 days and with Alizarin Red stained calcified areas after 21 days of osteogenic differentiation. Bar charts of the quantitative image analysis of alkaline phosphatase positive cells and Alizarin Red positive calcifications showing the time-dependent influence of Enoxaparin and Rivaroxaban on the osteogenic differentiation capacity of hMSC. Asterisks show significance levels of Dunn’s multiple comparison post-hoc tests to the control group (*n* = 5)

Alkaline phosphatase stainings of the differentiating MSCs of 5 donors on day 14 of osteogenic differentiation showed no visibly detectable changes in Enoxaparin- or Rivaroxaban-treated cells and untreated cells and quantitative image analysis of the stainings showed no changes in ALP intensity at all timepoints tested (Fig. 4). The decreased calcification capacity of Enoxaparin-treated cells shown by Alizarin staining on day 21 was easily detectable. Quantitative image analysis of the Alizarin staining data was conducted after 14 and 21 days and showed a significant reduction in calcification capacity at all Enoxaparin concentrations after 21 days of osteogenic differentiation (2 μg/ml: 67 %, 10 μg/ml: 67 %, 50 μg/ml: 65 %; *p* 0.031, 0.035, 0.049; respectively) while no changes were seen in Rivaroxaban-treated cells (Fig. 4).

## Discussion

Bone healing is a complex biological process that includes the stages of inflammation, repair and remodelling. The recruitment of MSCs, and their migration, proliferation and differentiation into osteogenic cells is essential for bone repair [17]. For postoperative clinical use, knowledge of the (negative) impact of drugs on bone healing is of great importance. In the context of VTE prophylaxis, reports show that long-term therapy with heparin and LMWH for the prevention of VTE following major orthopaedic surgery has an adverse effect on bone, with an increase in fractures and osteoporosis having been reported [4–7]. However, the specific effects of these drugs on the complex system of bone healing remain to be clarified.

We are the first to show that Enoxaparin increased the number of viable hMSCs during the first week of osteogenic differentiation. The similar growth capacities in both groups after 2 weeks of differentiation may be due to the osteoblast-like character of the cells, as the proliferation of mature osteoblasts is not influenced by Enoxaparin [18–20]. Solayar et al. showed that Enoxaparin treatment caused the down-regulation of osteoblast function that was associated with reduced mRNA expression of bone markers such as osteocalcin, Runx2 and BMP2 in mature osteoblasts [19]. Here, we show that these genes were affected as early as the first week of osteogenic differentiation, and that this was accompanied by a reduction in the gene expression of alkaline phosphatase, bone-specific transcription factor Osterix and the osteoblast-specific cadherin 11. Furthermore, we found that treatment with Enoxaparin drastically reduced the expression level of IGF2 and its binding protein two that have been shown to play pivotal roles during early osteogenic differentiation [21]. After 2 weeks of differentiation we found reduced expression levels of collagen type I, which is secreted by osteoblasts and is required for bone formation. As gene expression data may not accurately reflect the actual phenotype of the cell we also looked at the protein level and studied the expression of surface markers (CD10, CD49e, CD92) that had recently been reported to be highly regulated in MSC differentiation towards the osteogenic lineage [16]. The peptidase CD10 is speculated to participate in osteogenic differentiation by digestion of osteostatin, osteogenic growth peptide and calcitonin [22]. The fibronectin receptor CD49e, or integrin α5, is a key mediator of IGF2/IGFBP2 expression, involved in osteoblast adhesion and survival, and was recently found to trigger osteoblast differentiation and bone repair *in vivo* [21, 23, 24]. CD92 is responsible for choline uptake into the cell which is then incorporated into phosphatidylcholine mainly found in the lipid fraction of the calcification front during both intramembranous and endochondral bone formation [16]. In this study, we found for the first time that all three markers were down-regulated by Enoxaparin treatment in a dose- and time-dependent manner. In addition, we found a significant decrease in the calcification capacity of the Enoxaparin-treated differentiated hMSCs. Interestingly, Street et al found that bone repair in a rabbit model of fracture healing was attenuated by Enoxaparin and that biomechanical testing with torsional loading after 21 days revealed a significant reduction in strength, stiffness and energy absorbed to fracture [9]. In a rat model, Enoxaparin induced osteopenic changes and inhibition of bone formation. Furthermore, Casele et al. reported that in 16 women receiving Enoxaparin during pregnancy, bone density was significantly decreased at 6 months postpartum and 14 % of the patients showed bone loss of more than 10 % [25].

Since the publication of the four RECORD studies [3, 26–28], the use of Rivaroxaban increased to prevent VTE after orthopaedic surgery. However, its effect on bone healing is not yet understood in detail. A PubMed search (June 1^st^, 2015) using the keywords “Rivaroxaban” and “bone healing” found only four studies [11, 29–31] as did a search using the keywords “Rivaroxaban” and “osteoblast” [11, 19, 29, 31].

However, Marsell et al. revealed that examination of mature osteoblasts only reveals the effects during the late stage of bone formation initiated at 3–4 weeks after surgery [17].

Most studies focus on the effects on mature osteoblasts [19, 29, 31] but little is known about the initial phase of bone healing and the effect on the early stages in the first few weeks after surgery, which may be the more interesting part for daily clinical routine.

Therefore, we evaluated the proliferation and differentiation of hMSCs into osteoblasts during thromboprophylaxis, on which there have been no studies to date. While Gigi et al. found a Rivaroxaban dose-dependent reduction in the DNA synthesis of mature osteoblasts, Solayar et al. found no adverse effect on osteoblast viability [19, 29]. Both groups, however, proposed a significant influence on mature osteoblast function measured by a reduction of alkaline phosphatase activity in Rivaroxaban-treated osteoblasts that we, using differentiating hMSCs, only found during the first week of osteogenic differentiation when using the highest concentration of each drug. Furthermore, we found the calcification capacity of the differentiated hMSCs to be unaffected by Rivaroxaban. In addition, Rivaroxaban caused no down-regulation in the expression level of osteogenic marker genes or surface protein markers during the first 2 weeks of differentiation, but rather up-regulated IGF2, which triggers mesenchymal stromal osteogenic differentiation [21]. So, similar to a rat femur fracture model that showed no effect of Rivaroxaban on bone healing [11], in our study we were the first to show that Rivaroxaban has no inhibitory effects on the osteogenic differentiation of human mesenchymal stromal cells.

## Conclusions

The present study addressed the effects of the thromboprophylactic drugs Enoxaparin and Rivaroxaban on human mesenchymal stromal cells’ osteogenic differentiation in vitro. We showed for the first time that Enoxaparin promotes hMSC proliferation while inhibiting the expression of osteogenic marker genes and proteins as well as calcification. Rivaroxaban, however, had no effect on hMSCs during proliferation, osteogenic differentiation and calcification, pointing towards a lesser effect on the bone healing process.

To further understand the clinical relevance of the effects of VTE prophylaxis on postoperative bone healing, long term in vivo studies are needed.

### Ethical review committee statement

The study protocol was approved and authorized by the Institutional Review Board of the Heinrich-Heine-University Düsseldorf (study #3899).

## Additional files

Additional file 1: Figure S1. GAPDH Ct values show that there are no significant changes in the expression level of this gene over time and during drug treatment revealing that it can be used as housekeeping gene in this experimental set-up. (DOC 45 kb) Additional file 2: Table S1. Showing the primer sequences. (XLSX 9 kb)

## Abbreviations

ALP: alkaline phosphatase
BMP: bone morphogenetic protein
DMSO: dimethyl sulfoxide
hMSC: human mesenchymal stromal cells
IGF: insulin-like growth factor
IGFBP: insulin-like growth factor binding protein
LMWH: low-molecular-weight heparins
SEM: standard error of the mean
VTE: venous thromboembolism

## References

[1] Colwell CW (2007). Rationale for thromboprophylaxis in lower joint arthroplasty. Am J Orthod.

[2] Turpie AG (1991). Efficacy of a postoperative regimen of enoxaparin in deep vein thrombosis prophylaxis. Am J Surg.

[3] Eriksson BI, Borris LC, Friedman RJ, Haas S, Huisman MV, Kakkar AK (2008). Rivaroxaban versus enoxaparin for thromboprophylaxis after hip arthroplasty. N Engl J Med.

[4] Dahlman TC (1993). Osteoporotic fractures and the recurrence of thromboembolism during pregnancy and the puerperium in 184 women undergoing thromboprophylaxis with heparin. Am J Obstet Gynecol.

[5] Rajgopal R, Bear M, Butcher MK, Shaughnessy SG (2008). The effects of heparin and low molecular weight heparins on bone. Thromb Res.

[6] Squires JW, Pinch LW (1979). Heparin-induced spinal fractures. JAMA.

[7] Wawrzynska L, Tomkowski WZ, Przedlacki J, Hajduk B, Torbicki A (2003). Changes in bone density during long-term administration of low-molecular-weight heparins or acenocoumarol for secondary prophylaxis of venous thromboembolism. Pathophysiol Haemost Thromb.

[8] Demirtas A, Azboy I, Bulut M, Ucar BY, Alabalik U, Necmioglu NS (2013). Investigation of the effects of Enoxaparin, Fondaparinux, and Rivaroxaban used in thromboembolism prophylaxis on fracture healing in rats. Eur Rev Med Pharmacol Sci.

[9] Street JT, McGrath M, O'Regan K, Wakai A, McGuinness A, Redmond HP (2000). Thromboprophylaxis using a low molecular weight heparin delays fracture repair. Clin Orthop Relat Res.

[10] Kapetanakis S, Nastoulis E, Demesticha T, Demetriou T (2015). The effect of Low molecular weight heparins on fracture healing. Open Orthopaedics J.

[11] Kluter T, Weuster M, Bruggemann S, Menzdorf L, Fitschen-Oestern S, Steubesand N (2015). Rivaroxaban does not impair fracture healing in a rat femur fracture model: an experimental study. BMC Musculoskelet Disord.

[12] Ellensen VS, Abrahamsen I, Lorens J, Jonung T (2014). Effects of enoxaparin and dalteparin on proliferation and migration of patient-derived vascular smooth muscle cells. VASA Zeitschrift fur Gefasskrankheiten.

[13] Kubitza D, Becka M, Mueck W, Zuehlsdorf M (2007). Rivaroxaban (BAY 59-7939)--an oral, direct Factor Xa inhibitor--has no clinically relevant interaction with naproxen. Br J Clin Pharmacol.

[14] Mueck W, Borris LC, Dahl OE, Haas S, Huisman MV, Kakkar AK (2008). Population pharmacokinetics and pharmacodynamics of once- and twice-daily rivaroxaban for the prevention of venous thromboembolism in patients undergoing total hip replacement. Thromb Haemost.

[15] Livak KJ, Schmittgen TD (2001). Analysis of relative gene expression data using real-time quantitative PCR and the 2(-Delta Delta C(T)) Method. Methods.

[16] Graneli C, Thorfve A, Ruetschi U, Brisby H, Thomsen P, Lindahl A (2014). Novel markers of osteogenic and adipogenic differentiation of human bone marrow stromal cells identified using a quantitative proteomics approach. Stem Cell Res.

[17] Marsell R, Einhorn TA (2011). The biology of fracture healing. Injury.

[18] Birmingham E, Niebur GL, McHugh PE, Shaw G, Barry FP, McNamara LM (2012). Osteogenic differentiation of mesenchymal stem cells is regulated by osteocyte and osteoblast cells in a simplified bone niche. Eur Cell Mater.

[19] Solayar GN, Walsh PM, Mulhall KJ (2011). The effect of a new direct Factor Xa inhibitor on human osteoblasts: an in-vitro study comparing the effect of rivaroxaban with enoxaparin. BMC Musculoskelet Disord.

[20] Wagner ER, Luther G, Zhu G, Luo Q, Shi Q, Kim SH (2011). Defective osteogenic differentiation in the development of osteosarcoma. Sarcoma.

[21] Hamidouche Z, Fromigue O, Ringe J, Haupl T, Marie PJ (2010). Crosstalks between integrin alpha 5 and IGF2/IGFBP2 signalling trigger human bone marrow-derived mesenchymal stromal osteogenic differentiation. BMC Cell Biol.

[22] Ruchon AF, Marcinkiewicz M, Ellefsen K, Basak A, Aubin J, Crine P (2000). Cellular localization of neprilysin in mouse bone tissue and putative role in hydrolysis of osteogenic peptides. J Bone Miner Res.

[23] Kaabeche K, Guenou H, Bouvard D, Didelot N, Listrat A, Marie PJ (2005). Cbl-mediated ubiquitination of alpha5 integrin subunit mediates fibronectin-dependent osteoblast detachment and apoptosis induced by FGFR2 activation. J Cell Sci.

[24] Srouji S, Ben-David D, Fromigue O, Vaudin P, Kuhn G, Muller R (2012). Lentiviral-mediated integrin alpha5 expression in human adult mesenchymal stromal cells promotes bone repair in mouse cranial and long-bone defects. Hum Gene Ther.

[25] Casele HL, Laifer SA (2000). Prospective evaluation of bone density in pregnant women receiving the low molecular weight heparin enoxaparin sodium. J Matern Fetal Med.

[26] Kakkar AK, Brenner B, Dahl OE, Eriksson BI, Mouret P, Muntz J (2008). Extended duration rivaroxaban versus short-term enoxaparin for the prevention of venous thromboembolism after total hip arthroplasty: a double-blind, randomised controlled trial. Lancet.

[27] Lassen MR, Ageno W, Borris LC, Lieberman JR, Rosencher N, Bandel TJ (2008). Rivaroxaban versus enoxaparin for thromboprophylaxis after total knee arthroplasty. N Engl J Med.

[28] Turpie AG, Lassen MR, Davidson BL, Bauer KA, Gent M, Kwong LM (2009). Rivaroxaban versus enoxaparin for thromboprophylaxis after total knee arthroplasty (RECORD4): a randomised trial. Lancet.

[29] Gigi R, Salai M, Dolkart O, Chechik O, Katzburg S, Stern N (2012). The effects of direct factor Xa inhibitor (Rivaroxaban) on the human osteoblastic cell line SaOS2. Connect Tissue Res.

[30] Jameson SS, Rymaszewska M, Hui AC, James P, Serrano-Pedraza I, Muller SD (2012). Wound complications following rivaroxaban administration: a multicenter comparison with low-molecular-weight heparins for thromboprophylaxis in lower limb arthroplasty. J Bone Joint Surg.

[31] Somjen D, Katzburg S, Gigi R, Dolkart O, Sharon O, Salai M (2013). Rivaroxaban, a direct inhibitor of the coagulation factor Xa interferes with hormonal-induced physiological modulations in human female osteoblastic cell line SaSO2. J Steroid Biochem Mol Biol.

