# Supplementary material for: Effects of thromboprophylaxis on mesenchymal stromal cells during osteogenic differentiation: an in-vitro study comparing enoxaparin with rivaroxaban
Source: BMC Musculoskelet Disord. 2016 Mar 1;17:108. doi: 10.1186/s12891-016-0966-2 (PMC4772441; doi:10.1186/s12891-016-0966-2)

Supplemental Figure 1

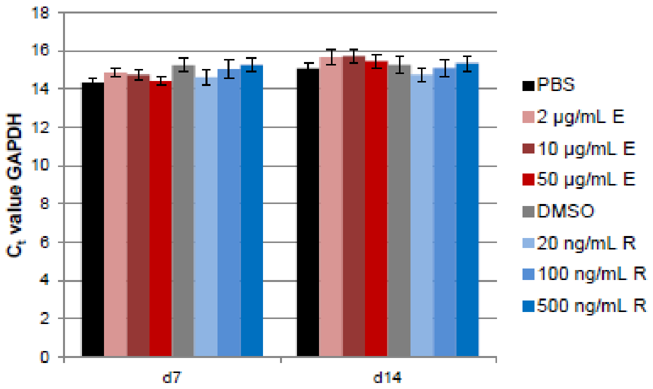

Supplement: Supplementary file 1 [file 12891_2016_966_MOESM1_ESM.doc]
